# A US‐Based Multi‐Site Pilot to Screen Hepatitis B Surface Antigen‐Positive Patients for Hepatitis D

**DOI:** 10.1111/jvh.14043

**Published:** 2024-12-12

**Authors:** Maggie Li, Bijou Hunt, Bindu Balani, Chinwe Ogedegbe, Peter Gordon, Joshua Hayden, Nancy Glick, Anita Chang, Su Wang, Mitchell Caponi, Lisa Yarber‐Cambron, Sandeep Bhat, Tyshea Ward, Madhu Suryadevara

**Affiliations:** ^1^ Sinai Infectious Disease Center, Sinai Chicago Chicago Illinois USA; ^2^ Department of Preventive Medicine Northwestern University Feinberg School of Medicine Chicago Illinois USA; ^3^ Hackensack University Medical Center Hackensack Meridian Health Hackensack New Jersey USA; ^4^ Hackensack Meridian School of Medicine Nutley New Jersey USA; ^5^ NewYork‐Presbyterian Queens/Columbia University Flushing New York USA; ^6^ Norton Healthcare Louisville Kentucky USA; ^7^ Asian Health Services Oakland California USA; ^8^ Department of Medicine, Cooperman Barnabas Medical Center Livingston New Jersey USA; ^9^ Family Health Centers at NYU Langone Health Brooklyn New York USA; ^10^ Infectious Disease/Family Treatment Center Newark Beth Israel Medical Center Newark New Jersey USA

**Keywords:** hepatitis B, hepatitis D, prevalence

## Abstract

Hepatitis D (HDV) is a severe infection with well‐recognised clinical ramifications that remains relatively neglected and underdiagnosed; consequently, the epidemiology of HDV is poorly characterised, both in the United States and globally. In 2022, a pilot project involving eight healthcare institutions was undertaken to ascertain the prevalence of HDV in healthcare institutions with an HBV seropositivity of at least 1%, describe the characteristics of patients testing positive for HDV, and evaluate diagnostic and laboratory processes of HDV screening. From August 2022 to April 2024, a total of 106,693 patients were tested for HBsAg, of whom 65,341 (61.2%) were female and 40,863 (38.3%) were male, with a mean age of 47 years. The overall HBsAg positivity rate was 1.04% (*n* = 1112). Among the HBsAg+ samples, 645 (58.0%) underwent HDV Ab testing. The HDV Ab positivity rate was 0.81% (*n* = 9), with 2 cases of HDV RNA positivity (0.18%). The incomplete testing reflects several challenges associated with screening for both HBV and HDV. Further research is necessary to better understand the epidemiology and burden of HDV in the United States and considerations for implementation.

## 1 Introduction

Hepatitis D (HDV) is a ‘defective’ virus, or viroid, that requires the hepatitis B surface antigen (HBsAg) to undergo replication [1] in a life cycle that remains poorly understood. Given its vital dependence on HBsAg, HDV transmission can only occur in conjunction with hepatitis B virus (HBV) infection through two primary modalities: simultaneous infection with both HBV and HDV or superinfection of an individual chronically infected with HBV [2].

The clinical expression of HDV is subject to the modality of infection. Coinfection can result in a range of hepatic outcomes, from mild to severe fulminant hepatitis [3]. In contrast, superinfection typically leads to severe acute hepatitis, resulting in worsened hepatic necroinflammation and an increased risk of progression to cirrhosis and hepatocellular carcinoma [4]. More than 90% of patients with superinfection progress to chronic HDV, which is considered to be the most aggressive form of chronic viral hepatitis [5], as it exacerbates the preexisting HBV‐related liver damage [6].

Despite the severe clinical ramifications of HBV/HDV dual infection, HDV remains relatively neglected and underdiagnosed, both in the United States and globally. While accurate country‐specific and global prevalence estimates of HDV remain elusive [7], studies suggest a US seropositivity prevalence of 0.11% [8] and a global seropositivity prevalence of 0.16% [4]. The epidemiology of HDV continues to evolve and is believed to be influenced by various factors. These include shared risk factors for human immunodeficiency virus (HIV) and hepatitis C virus (HCV), such as men who have sex with men, injection drug use and commercial sex work [4], as well as local prevalence and immigration patterns [9]. Successful HBV vaccination efforts may have contributed to decreases in HDV prevalence in high‐income countries [10], but the epidemiology of HDV in many parts of the world remains poorly characterised due to a lack of screening and surveillance systems [4].

Numerous researchers advocate for universal HDV screening among patients with chronic HBV, citing the severity of dual infection, the evolving and incompletely understood epidemiology, and the advent of promising therapeutic interventions and prevention strategies [4, 8, 10, 11, 12, 13, 14, 15, 16]. The adoption of universal HDV screening, however, has remained suboptimal. For instance, a study examining HDV screening rates among US veterans with chronic HBV found that screening occurred in fewer than 8% of this population, likely attributable to inexperience with HDV, insufficient training of providers regarding high risk groups, infrequent referrals to specialists, and poor access to HDV screening [17]. Complex diagnostic pathways, as well as the scarcity of highly effective therapeutics, also contribute to the low screening and treatment rates [16].

Nevertheless, accurate HDV seroprevalence data are essential for informing prevention strategies and treatment programs, thereby enhancing the care continuum for impacted patients. In response to this pressing need, a multisite pilot project was launched to advance the understanding of HDV epidemiology in the United States. This paper describes the diagnostic and laboratory procedures of the project sites and presents data on patients tested for HBV and HDV.

## 2 Methods

### 2.1 Programme Overview

Since 2010, Gilead Sciences Inc.'s (Gilead Sciences) Frontlines of Communities in the United States (FOCUS) programme has supported HIV, HCV, and HBV testing, along with linkage to care initiatives, across various institutions in 34 states and 2 countries. In 2022, Gilead Sciences' Government Affairs and Medical Affairs divisions launched the HDV Screening and Linkage to Care (SLTC) pilot project. Eight sites were selected to participate based on specific criteria, including a minimum HBV seropositivity rate of 1%, a minimum volume of 4000 HBV tests per year, the existence of an HBV routine SLTC process, a proven track record in dissemination, experience in serving impacted patient populations, and geographic areas with a suspected HDV burden. As a collaborative, these sites would develop and share best practices in routine HDV SLTC in alignment with local, state and national public health guidelines. The objectives of the HDV SLTC pilot were multifaceted. Specially, we aimed to 1) ascertain the prevalence of HDV in high‐burden HBV areas across the United States, 2) describe the characteristics of patients testing positive for HDV, 3) provide an overview of diagnostic and laboratory processes, 4) discuss HDV SLTC, and 5) discuss HDV surveillance, data, guidelines and policy. This paper focuses on Goals 1–3 and includes the use and discussion of HDV serologic assays that are not currently approved by the Food and Drug Administration (FDA).

### 2.2 Setting

The eight sites included in the HDV SLTC pilot are shown in Table 1. The sites are all located in urban/suburban areas across the contiguous United States (California (*n* = 1); Illinois (*n* = 1); Kentucky (*n* = 1); New Jersey (*n* = 3); and New York (*n* = 2)). While the primary populations served by the sites varied both in terms of race/ethnicity and socio‐economic status, all sites served large immigrant and/or injection drug use populations.

**TABLE 1:** Characteristics of pilot sites, 2022.

Name	Location	Setting	Patient populations	Data collection period	Partner lab
Asian Health Services	Oakland, California	Urban	Asian, uninsured or publicly insured, immigrants, ~75% non‐English speaking	1 August 2022 to 30 June 2023	Quest Diagnostics
Cooperman Barnabas Medical Center	Livingston, New Jersey	Urban and sub‐urban	Black/African American, White, Hispanic/Latinx, Asian Pacific Islander, immigrants, mixed income	1 August 2022 to 31 August 2023	LabCorp
Family Health Centers at NYU Langone	Brooklyn, New York	Urban	Hispanic/Latinx, Black/African American, Asian Pacific Islander, immigrants, low‐income, LGBTQ+	1 September 2022 to 30 April 2024	ARUP Laboratories
Hackensack Meridian Medical Center (HUMC and PMC)	Hackensack, New Jersey	Urban	Hispanic/Latinx, Black/African American, Asian Pacific Islander, immigrants, low‐income	1 January 2022 to 31 March 2024	Quest Diagnostics
Newark Beth Israel Medical Center	Newark, New Jersey	Urban	Hispanic/Latinx, Black/African American, Asian Pacific Islander, immigrants, low‐income, LGBTQ+	1 October 2022 to 30 September 2023	LabCorp
NewYork‐Presbyterian Queens	Flushing, New York	Urban	Hispanic/Latinx, Asian, Black/African American, immigrants, low‐income	Site unable to provide data	ARUP Laboratories
Norton Healthcare	Louisville, Kentucky	Urban	White, Black/African American, low‐income, injection and non‐injection drug use, foreign‐born residents, LGBTQ+	1 November 2023 to 30 April 2024	ARUP Laboratories
Sinai Health System	Chicago, Illinois	Urban	Hispanic/Latinx, Black/African American, Asian, immigrants, low‐income	15 April 2022 to 30 April 2024	Alverno Laboratories

**FIGURE 1:**
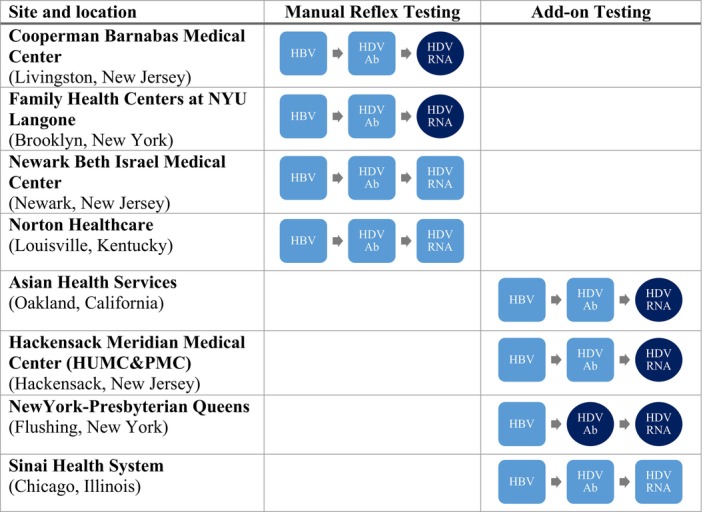
Laboratory processes of pilot sites, 2022. 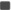, initial sample; 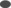, second sample; HBV, hepatitis B surface antigen positive; HDV Ab, hepatitis delta virus antibody; HDV RNA, hepatitis delta virus ribonucleic acid.

Sites fell into two distinct categories when considering their laboratory processes: Manual reflex testing (Cooperman Barnabas Medical Center, Family Health Centers at NYU Langone, Newark Beth Israel Medical Center, Norton Healthcare) and Add‐on testing (Asian Health Services, Hackensack Meridian Medical Center, NewYork‐Presbyterian, Sinai Chicago) (Figure 1). Manual reflex testing involved workflows where lab personnel played a key role in moving samples through testing steps. While these processes could be loosely considered reflex testing, they lacked the important characteristic of being fully automated [18]. At sites with a manual reflex testing model, lab personnel were integral in steps such as identifying HBsAg+ samples, locating and placing correct orders for subsequent HDV Ab and/or HDV RNA testing, and pulling, preparing and shipping samples out to external labs where applicable. In contrast, add‐on testing involved workflows where care team members (physicians, navigators, and data specialists) were the lead agents in moving samples through the testing steps. At sites with an add‐on testing model, care team members were responsible for identifying HBsAg+ patients (usually through a daily report reviewed by a team member). They then communicated the need for HDV Ab and/or HDV RNA add‐on testing to the lab, either by placing an order in the electronic medical record (EMR) or contacting the lab via phone or fax.

### 2.3 Data Collection

Data collection periods differed across sites (Table 1). However, all sites that were able to collect and report data did so internally, sharing aggregated de‐identified data, which were then combined to produce cross‐collaborative totals.

The following sites obtained IRB approval: Hackensack Meridian Medical Center (Hackensack Meridian Health Research Institute, IRB# Pro2023‐0249), Sinai Chicago (The Mount Sinai Hospital Institutional Review Board, IRB# MSH‐IRB‐24‐20) and Norton Healthcare (Norton Research Institute, IRB# 1371208). In some cases, this approval was a reflection of the decision that this work did not constitute human subject research. Five sites (Asian Health Services, Cooperman Barnabas, Family Health Centers at NYU Langone, Newark Beth Israel Medical Center and NewYork‐Presbyterian Queens) did not obtain IRB approval as their institution did not deem this to constitute human subject research.

## 3 Results

Collectively, the collaborative performed HBsAg testing in a total of 106,693 unique patients. Patient characteristics are summarised in Table 2, with a mean age of 47 years. Among them, 61.2% were female and 38.3% were male. The overall HBsAg positivity rate was 1.04% (*n* = 1112). Among HBsAg+ samples, 58.0% were tested for HDV Ab. The HDV Ab positivity rate was 0.81% (*n* = 9), with two cases of HDV RNA positivity (0.18%). While the majority of HBsAg testing was performed among persons of Hispanic/Latino ethnicity (29.4%) and those identifying as non‐Hispanic Black (27.8%), the largest portion of HBsAg+ results was observed among persons identifying as non‐Hispanic Asian (35.3%). HDV Ab+ results were observed across all racial/ethnic groups: non‐Hispanic White and Hispanic/Latino (11.1%, *n* = 1, respectively); non‐Hispanic Black (22.2%, *n* = 2); and non‐Hispanic Asian (55.6%, *n* = 5). HDV RNA+ results (*n* = 2) were observed among non‐Hispanic White and non‐Hispanic Black (50%, *n* = 1, respectively). While the majority of HBsAg testing was performed among females (61.2%), a slightly higher proportion of HBsAg+ results occurred among males (51.3%).

**TABLE 2:** Patient characteristics and testing outcomes.

	HBsAg tested	HBsAg‐positive		HDV tested	HDV Ab‐positive		HDV RNA‐positive	
	*n* (%)	*n* (%)	Prevalence	*n* (%)	*n* (%)	Prevalence	*n* (%)	Prevalence
Race/Ethnicity								
Non‐Hispanic White	23,197 (21.7)	118 (10.6)	0.51	39 (6.0)	1 (11.1)	0.85	1 (50.0)	0.85
Non‐Hispanic Black	29,712 (27.8)	288 (25.9)	0.97	88 (13.6)	2 (22.2)	0.69	1 (50.0)	0.35
Non‐Hispanic Asian	7496 (7.0)	393 (35.3)	5.24	359 (55.7)	5 (55.6)	1.27	0 (0.00)	0.00
Hispanic/Latino	31,386 (29.4)	107 (9.6)	0.34	30 (4.7)	1 (11.1)	0.93	0 (0.00)	0.00
Other/Unknown	14,902 (14.0)	206 (18.5)	1.38	128 (19.8)	0 (0.00)	0.00	0 (0.00)	0.00
Sex								
Male	40,863 (38.3)	570 (51.3)	1.39	348 (54.0)	5 (55.6)	0.88	2 (100.0)	0.35
Female	65,341 (61.2)	541 (48.7)	0.83	297 (46.0)	4 (44.4)	0.74	0 (0.00)	0.00
Other/Unknown	488 (0.5)	1 (0.1)	0.20	0 (0.0)	0 (0.00)	0.00	0 (0.00)	0.00
Age (mean)	47.2	50.1		51.6	44.0		40.0	
Total patients tested	106,693	1112 (1.04)	1.04	645 (58.0)	9 (1.40)	0.81	2 (22.22)	0.18

A total of nine HDV Ab+ results were identified, with two of these also being RNA+. The first HDV Ab+ and RNA+ patient was a non‐Hispanic White male, aged 49, from Ukraine. This patient, who had no coinfection with HCV or HIV and was a current tobacco user, was tested for HBV during refugee screening. The viral load at the time of HDV diagnosis was 68,500. The other HDV Ab+ and RNA+ patient was a non‐Hispanic Black male, aged 31, from Eritrea. He also had no coinfection with HCV or HIV and had no history of substance use. His viral load at HDV diagnosis was > 5,800,000.

Regarding HDV Ab+ patients, one was a non‐Hispanic Black male, aged 68, with a past medical history of HBV and intravenous drug use (IVDU). He had been on HBV treatment but had discontinued it and had not been tested for HDV RNA. The second HDV Ab+, but RNA−, patient was a 52‐year‐old female from the Philippines. She had undergone a deceased donor renal transplant and had a history of chronic hepatitis B. The third HDV Ab+, but RNA−, patient was a 56‐year‐old female from Afghanistan, who had no coinfection with HCV or HIV and no history of substance use. The fourth HDV Ab+, but RNA−, patient was a 50‐year‐old male from China, who had a reactive HCV Ab with undetectable HCV RNA, no coinfection with HIV, and no history of substance use. The fifth HDV Ab+ patient, an 83‐year‐old female from China, could not have her RNA status determined as she was lost to follow‐up. The sixth HDV Ab+, but RNA−, patient was a 53‐year‐old male from China with a history of prior cocaine use. Labs showed the patient had previously cleared HCV infection and was HIV‐negative. The last HDV Ab+, but RNA−, patient was a 53‐year‐old Hispanic female with an unknown country of origin, no coinfection with HCV or HIV, and no history of substance use.

## 4 Discussion

HDV infection can occur in patients with HBV and, when present, tends to lead to increased morbidity and mortality of the disease [11]. HDV screening has been recommended for patients with HBV, though variations exist in guidelines regarding universal (screening all patients with HBV) [19] versus risk‐based (screening patients with high‐risk behaviours and those from endemic countries) [20] approaches. Our pilot tested a total of 106,693 patients for HBsAg, identifying 1112 HBsAg+ patients, among whom nine cases were found to be HDV Ab+ (0.81%) and two cases were found to be HDV RNA+ (0.18%). Our findings for HDV prevalence differed from another study, which reported a 6% HDV Ab prevalence and a 1.87% HDV RNA prevalence among chronic hepatitis B patients [15]. However, our study included patients with and without known chronic hepatitis B infection, which may explain the lower HDV prevalence. In addition, despite employing a universal HDV screening approach for HBsAg+ patients, only 58.0% of HBsAg+ patients underwent HDV Ab testing, which could further account for the lower prevalence observed. The incomplete testing reflects several challenges associated with screening for both HBV and HDV, as reported by our sites.

### 4.1 Challenges

Screening for HBsAg, crucial for identifying candidates for HDV screening, was often incomplete due to providers' lack of education regarding HBV screening criteria. This is evident at our sites, where more female patients (61.2%) were tested for HBV than male patients (38.3%), possibly because female patients underwent pregnancy screenings. However, HBV screening criteria are not exclusive to female individuals; the Centers for Disease Control and Prevention recommends that all adults aged 18 and older be screened for HBV at least once in their lifetime [21]. Had we tested more males, we might have identified more HBsAg+ males, leading to increased HDV testing and a higher number of HDV+ cases. Thus, improving providers' awareness of HBV screening criteria is essential for achieving comprehensive screening practices across all patient demographics.

Likewise, screening for HDV was hindered by the variety of HDV test options in the EMR, leading to instances of incorrect test orders. In addition, sites faced challenges regarding sample availability for HDV screening. Some samples were discarded upon HBsAg positivity identification, while others were accessible but insufficient for additional testing. At sites where an additional tube was required for confirmatory testing, this introduced potential for loss‐to‐follow‐up that could be avoided if only one tube was required for all tests to be completed. Overall, the absence of HDV automatic reflex testing in general posed a risk of workflow disruptions and potential delays. This deficiency may have resulted in instances where HBsAg+ patients were not screened for HDV, potentially missing crucial diagnostic opportunities. This pilot had an overall completion rate of 58% for testing HBsAg+ samples for HDV Ab and a 67% completion rate for testing HDV Ab+ samples for HDV RNA. For sites in this pilot, all of which lacked an automatic reflex process, the burden was placed on staff to manually ensure that HBsAg+ samples underwent HDV screening.

### 4.2 Modifications to EMR Systems

To improve the screening efficacy for HDV in HBsAg+ patients, we recommend modifications to EMR systems. These include incorporating specific instructions within the testing order regarding the required blood volume and collection tube type. This will help minimise potential errors during sample collection. Furthermore, we propose integrating available HDV tests into the EMR (as is current practices for routine HIV screening [22]) and establishing standardised order sets that bundle relevant tests. This approach helps prevent the ordering of incorrect tests. Most importantly, laboratories are encouraged to consider implementing double reflex confirmatory testing, as is best practice for hepatitis C testing [23].

### 4.3 Reflex Testing

Double reflex testing involves following up all HBsAg+ samples with HDV Ab testing, and subsequently performing HDV RNA testing on those samples positive for HDV Ab [24]. This process, triggered by a positive HBsAg result, facilitates the immediate ordering of HDV Ab testing, which can eliminate manual tracking of HBsAg+ patients requiring HDV testing and reduces the need for add‐on HDV testing requests. Most importantly, double reflex testing addresses the limitations of the risk‐based screening approach, which has proven insufficient for detecting HDV+ patients. While our project did not identify HDV cases that would have been missed with a risk‐based approach, a recent study found that the risk‐based screening approach would miss 18% of HDV+ patients due to unreported risk factors [15]. Thus, this double reflex approach has gained support and may become more feasible with anticipated updates to FDA‐approved screening (Ab) and diagnostic tests (RNA) and advancements in sample collection capabilities to support this sequential testing cascade.

### 4.4 Limitations

This pilot project had several aims, including to ascertain the prevalence of HDV in eight healthcare institutions with an HBV seropositivity of at least 1% within the United States. However, due to significant challenges related to implementation, we caution against these estimates being used to inform policy related to HDV testing. Future studies are needed to esbtablish protocols that clearly delineate testing processes, ensuring a high rate of completion along the cascade, and should be carried out in settings that can ensure adherence to such a testing protocol.

While the prevalence findings from this project may not be generalisable, the sites were able to contribute considerable learnings on how to implement HDV screening. Sites were diverse in several key areas, including geography, patient population served, institutional hierarchy, laboratory processes, and primary payor sources. Each site employed a methodology they believed would work for their institution, taking into account factors such as labs, staffing, patient population, providers, C‐suite, and risk factors,and all came across similar but different challenges. Understanding and preparing for these challenges allows future sites to better plan for the implementation of HDV screening and potentially avoid similar pitfalls.

## 5 Conclusion

The prompt identification of HDV is crucial for improving patient outcomes, enabling clinicians to target treatment for those most likely to benefit once it becomes widely available. However, implementing HDV screening involves various considerations, including the appropriateness of screening for specific populations, the availability of reflex testing capabilities within laboratory infrastructure, and unique workflows. The findings of this pilot underscore the challenges in accurately determining the prevalence of HDV in both US healthcare institutions specifically and high‐burden HBV areas generally. However, accurate HDV epidemiological data are essential for informing prevention strategies and developing tailored treatment programmes, thereby enhancing the care continuum for impacted patients. Further research is necessary to better understand the epidemiology and burden of HDV.

## Conflicts of Interest

The authors declare no conflicts of interest.

## Data Availability

The data that support the findings of this study are available from the corresponding author upon reasonable request.
